# Editorial: Advances in Pathogenesis, Etiology, and Therapies for Ankylosing Spondylitis

**DOI:** 10.3389/fimmu.2021.822582

**Published:** 2021-12-23

**Authors:** Chih-Wei Chen, James Cheng-Chung Wei, Jieruo Gu, David Yu

**Affiliations:** ^1^ Institute of Medicine, Chung Shan Medical University, Taichung, Taiwan; ^2^ Department of Allergy, Immunology & Rheumatology, Chung Shan Medical University Hospital, Taichung, Taiwan; ^3^ Graduate Institute of Integrated Medicine, China Medical University, Taichung, Taiwan; ^4^ Rheumatology Department of the Third Affiliated Hospital of Sun Yat-sen University, Guangzhou, China; ^5^ Department of Medicine, University of California, Los Angeles, CA, United States

**Keywords:** ankylosing spondylitis, acquired immunity, diagnosis, innate immunity, therapy

Ankylosing spondylitis (AS) is a chronic inflammatory rheumatic disease caused by the disrupted balance of both the innate immune system and acquired immune system in response to environmental factors (1). AS causes inflammatory back pain and affects the spine and sacroiliac joints, which can lead to a drop in life quality of patients, as well as an increased burden to patients and society (2). In recent years, a growing number of studies have been conducted to investigate the pathogenesis and etiology, imaging techniques, and treatment in AS (3–7). In this Research Topic, there are three review articles and seven research articles published, mainly focusing on pathogenesis and etiology, diagnosis and therapies, and related assessment tools of AS.

Hong et al. investigated the genetic association between IL 6 and autoimmune arthritis using multiple genome-wide association studies (GWAS) datasets, and they found a genetic association between the increased level of IL-6 signalling and risk of RA and AS, as well as observed the sexual difference in IL6-intermediate susceptibility to autoimmune arthritis. Controversial results on the effect of infections on the risk of AS were reported by previous studies, which is quantitatively investigated by Zhang et al. through a meta-analysis. They confirmed that the risk of AS can be significantly enhanced by infections, such as infections with adjusted comorbidities, viral infection. Liu et al. further conducted a meta-analysis to clarify the alteration of the immune system in patients with AS. They found that the pathogenesis of AS can be ascribed to the disequilibrium between Th17 and Tregs, Th1 and Th2, which further supports that AS is resulted from the disrupted balance of the innate immune system and acquired immune system (8).

The diagnosis is the key to reducing the burden on patients and society caused by AS. Tu et al. employed the MRI images to identify non-radiographic axial spondyloarthritis (nr-axSpA) and radiographic axial spondyloarthritis (r-axSpA), the latter of which is also known as AS. They found that AS patients presented more active inflammatory and chronic structural damages, while erosion was more frequently observed in MRI of nr-axSpA patients. Han et al. systematically analysed clinical and imaging hip data to examine hip changes in AS patients by MRI and X-ray. They observed that more than 40% of AS patients with minimal or no hip pain had hip changes, which can be used for early diagnosis.

Various therapies for AS have been studied, such as tofacitinib – an oral Janus kinase inhibitor (9) and Risankizumab – an IL-23 inhibitor (10). An increasing number of studies found that cytokine signalling *via* the IL-17A pathway was a major factor in the pathogenesis of spondyloarthritis (SpA) (11, 12). Tok et al. assessed the influence of inhibition of RAR related orphan receptor-g (RORC) on experimental SpA in HLA-B27 transgenic (tg) rats, and they found that experimental SpA in the HLA-B27 tg rat model could be accelerated and aggravated by RORC inhibitor treatment. Wang and Maksymowych reviewed recent studies on the role of the IL-23/IL-17 pathway in the pathophysiology of inflammation to discuss the treatment of AS. They found that the inhibition of IL-17 cytokines contributed to the inflammatory symptom control in patients with axSpA, but the IL-23 blockade was ineffective in the treatment. Chen et al. employed the gut microbiome as a biomarker to evaluate the effectiveness of adalimumab therapy in AS patients, based on previous discoveries that the gut microbiome was associated with the initiation and development of AS, and their findings suggested the gut microbiome was restored by adalimumab therapy in AS patients and therefore could be used as a predictive tool for treatment response.

Moreover, assessment technologies have been investigated to support the diagnosis and therapeutic interventions. Han et al. studied the effect of therapeutic interventions that can be assessed by Micro-CT, and they found that Micro-CT could be used to quantitatively assess the extent of axial involvement in mouse spondylitis caused by proteoglycan aggrecan (PGA). Zhang et al. quantitatively assessed the effect of tumour necrosis factor (TNF)-a inhibitor treatment in patients with spondyloarthritis (SpA) through clinical and MRI assessment, which was suggested as an accurate evaluation tool to guide targeted treatment.

In conclusion, the papers collected in this Research Topic contributed to the understanding of the pathogenesis and etiology of AS, including hereditary and environmental factors, as well as the development of diagnosis and therapies, and related assessment technologies.

## Author Contributions

C-WC and JC-CW contributed equally to the writing and reviewing of the article. All authors contributed to the article and approved the submitted version.

## Conflict of Interest

The authors declare that the research was conducted in the absence of any commercial or financial relationships that could be construed as a potential conflict of interest.

## Publisher’s Note

All claims expressed in this article are solely those of the authors and do not necessarily represent those of their affiliated organizations, or those of the publisher, the editors and the reviewers. Any product that may be evaluated in this article, or claim that may be made by its manufacturer, is not guaranteed or endorsed by the publisher.
